# Organoids as an emerging platform for osteoarthritis drug development

**DOI:** 10.1002/ctm2.70806

**Published:** 2026-09-03

**Authors:** Xukun Lyu, Jianhua Wang, Jiacan Su

**Affiliations:** ^1^ Department of Orthopedics Xinhua Hospital Affiliated to Shanghai Jiao Tong University School of Medicine Shanghai China; ^2^ Trauma Orthopedics Center Xinhua Hospital Affiliated to Shanghai Jiao Tong University School of Medicine Shanghai China; ^3^ Institute of Musculoskeletal Injury and Translational Medicine of Organoids Xinhua Hospital Affiliated to Shanghai Jiao Tong University School of Medicine Shanghai China

## 1 THE MODELLING GAP IN OSTEOARTHRITIS DRUG DEVELOPMENT

**FIGURE 1:**
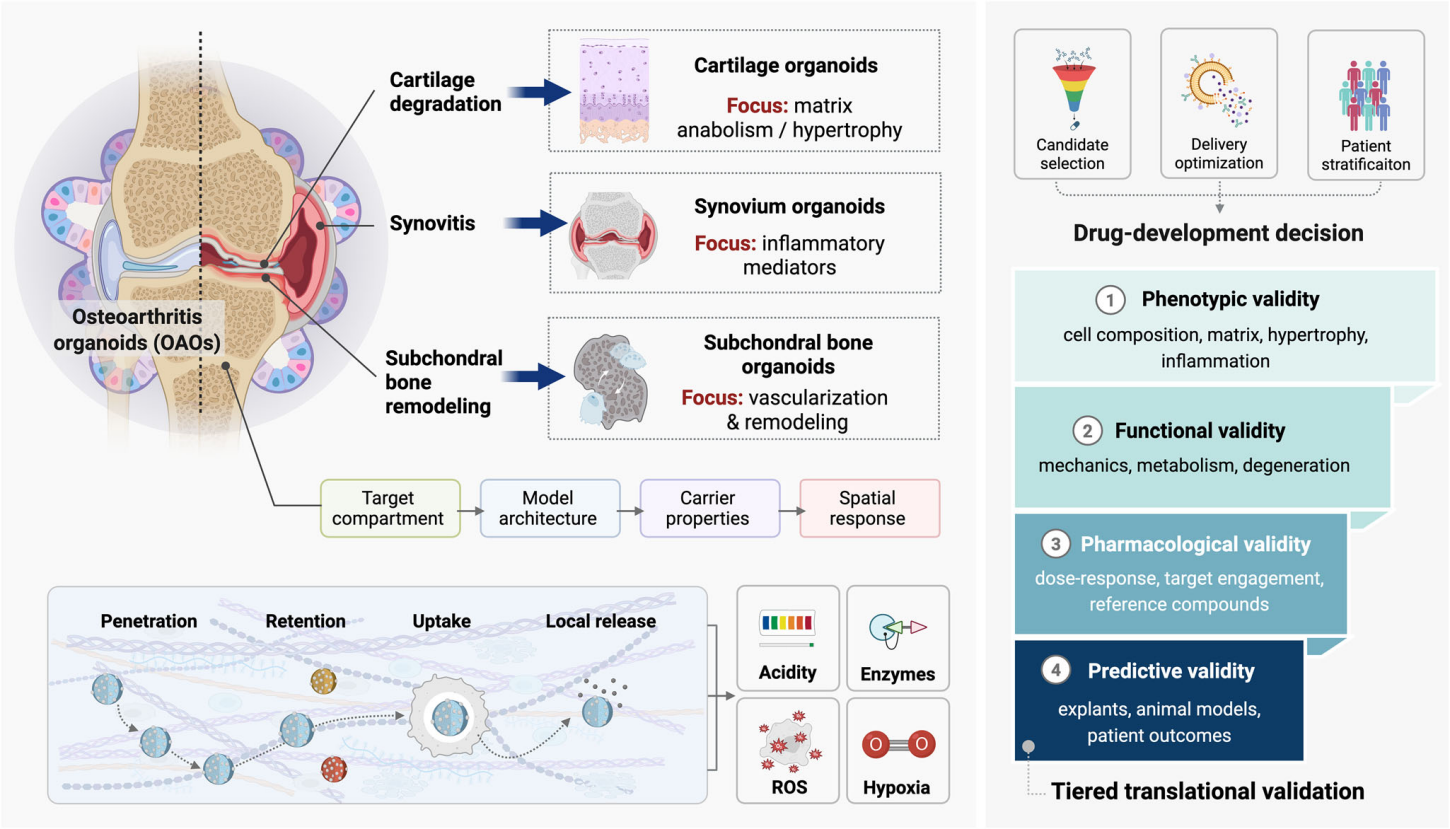
Organoid‐guided osteoarthritis drug development. Compartment‐specific cartilage, synovial and subchondral bone organoids model distinct osteoarthritis (OA) features and guide carrier design and spatial drug responses. Candidates then undergo tiered phenotypic, functional, pharmacological and predictive validation to inform candidate selection, delivery optimisation and patient stratification. Created in BioRender.com.

Osteoarthritis (OA) is a whole‐joint disorder involving cartilage, synovium, subchondral bone, immune cells and mechanical forces.^1^ Drug development is therefore constrained by more than target identification. Candidate therapies must reach the relevant joint compartment, remain active within a dense extracellular matrix and produce durable tissue‐level benefits.^2^ Conventional two‐dimensional cell cultures support rapid screening but poorly reproduce cell‐matrix and multicellular interactions. Explants preserve native architecture but have limited availability, culture duration and experimental flexibility. Animal models provide systemic exposure and functional outcomes, yet species differences and the acute nature of many induced models limit their correspondence with chronic OA. Organoids may help bridge this modelling gap. The term OA organoids (OAOs) refers to a self‐organising, three‐dimensional human‐cell system that reproduces defined aspects of joint‐tissue composition, architecture and function.^3^ This definition distinguishes organoids from simple spheroids, scaffold‐engineered cartilage constructs and native explants. The most defensible role of OAOs is as an intermediate platform that connects human‐relevant disease modelling with controlled perturbation, mechanistic screening and local drug‐delivery assessment (Figure 1).

## 2 ENGINEERING OSTEOARTHRITIS ORGANOIDS FOR MECHANISM‐GUIDED DRUG DESIGN

Mechanism‐guided therapeutic design requires two linked decisions: which joint compartment to model and which therapeutic function to optimise. Model selection should therefore begin with the anatomical target. Cartilage organoids are most relevant to matrix anabolism, catabolism and hypertrophic differentiation; synovial or immune components are needed for anti‐inflammatory strategies; and subchondral bone models are required for therapies directed at vascularisation, bone remodelling or hypoxic homeostasis. This principle is illustrated by a double‐reporter cartilage organoid platform that screened 2040 approved drugs and identified the α‐adrenergic receptor antagonist phentolamine as a promoter of chondrogenesis and suppressor of hypertrophy. Subsequent validation in explants and animal models showed how organoids can improve mechanistic resolution before candidates advance to higher‐order models.^4^

Once the relevant compartment has been selected, OAOs can be engineered to reproduce the barriers that determine local drug exposure. Diseased cartilage is avascular and contains a dense, negatively charged extracellular matrix, making carrier transport highly dependent on size, surface charge, ligand density and release kinetics. Cartilage organoids constructed with silk fibroin‐DNA double‐network hydrogels provide one route to recreating this environment.^5^ Extending this strategy, 3D‐bioprinted piezoelectric cartilage‐bone organoids combine compartment‐specific bioinks with mechanically induced bioelectrical cues to recreate chondral and osseous niches and promote tissue maturation.^6^ By controlling glycosaminoglycan content, collagen organisation, tissue scale and interfacial structure, such models could compare drug penetration, retention, cellular uptake and spatial distribution. This step links tissue architecture to the physicochemical design of the delivery system.

The next step is to determine whether delivery remains effective within the spatially heterogeneous OA microenvironment. Acidity, reactive oxygen species, enzymatic activity and hypoxia vary across disease compartments and over time. Reconstructing these gradients in organoids could test whether a responsive formulation releases its payload at the intended anatomical site, engages its target and avoids local tissue toxicity. The molecular target should also guide the required tissue distribution. Therapies directed at subchondral bone should extend beyond cartilage‐surface retention to achieve effective exposure within the subchondral compartment and modulate osteochondral crosstalk. For example, genetic deletion of *Lcp1* or pharmacological inhibition of L‐plastin suppresses osteoclastogenesis and type‐H angiogenesis, preserves joint hypoxia and chondrocyte HIF‐1α activity, and delays OA progression.^7^ Together, these steps connect target compartment, model architecture, carrier behaviour and functional response. They also define the evidence that must be established before an organoid‐guided candidate enters translational validation.

## 3 OSTEOARTHRITIS ORGANOIDS FOR PRECLINICAL DRUG VALIDATION

Translational validation should begin with a clearly defined context of use. An OAO intended for mechanistic screening, delivery optimisation, or patient stratification requires different performance criteria. Four levels of validity provide a staged framework. Phenotypic validity establishes reproducible cell composition, extracellular matrix, inflammatory responses and hypertrophic state. Functional validity confirms relevant mechanical, metabolic or degenerative behaviour. Pharmacological validity then tests interpretable dose‐response relationships, target engagement and concordant ranking of reference compounds. Finally, predictive validity compares organoid responses with human explants, appropriate animal models and, ultimately, patient outcomes. Progression through these levels converts biological resemblance into evidence that is relevant to a specific drug‐development decision. Each level should be supported by multidimensional readouts rather than viability alone. Spatial exposure, matrix synthesis and degradation, inflammatory mediators, lineage composition, mechanical function and local tissue toxicity together link delivery to mechanism and tissue response.^8^ Patient‐derived organoids could extend this framework by capturing interindividual variation and supporting functional precision medicine.^9^ In OA, however, their predictive value must be tested across clinically relevant differences in inflammation, mechanical loading, genetic susceptibility and subchondral bone remodelling. Analytical validity, clinical validity and clinical utility are therefore required before organoid‐derived phenotypes can guide treatment. Local OAOs also cannot determine systemic toxicity or whole‐body pharmacokinetics, which still require complementary multi‐organ, animal or clinical evidence.

## 4 LIMITATIONS AND OUTLOOK

Current OAOs still have limited capacity to recapitulate complex multicompartment interactions and long‐term mechanical cues. Variability in culture and maturation conditions may also affect reproducibility and drug response. Standardisation should therefore define identity, purity, stability, matrix composition, hypertrophic propensity, compressive properties, inflammatory responsiveness and osteochondral coupling, together with donor‐level and batch‐level reproducibility. Multi‐omics, high‐content imaging and label‐free phenotyping may strengthen this framework, while computational analysis may integrate patient information, model construction, drug exposure and multimodal readouts.^10^ Such tools will be valuable only if their outputs remain interpretable and externally validated. OAOs are thus best viewed as an emerging component of a staged evidence pathway: they can refine mechanistic and delivery decisions in a human‐relevant setting, but their translational value depends on rigorous qualification and confirmation in complementary models.

## CONFLICT OF INTEREST STATEMENT

The authors declare no conflict of interest.
